# 

**Published:** 1867-05

**Authors:** 


### Books and Pamphlets Received.

The Science and Practice of Medicine, by William Aitken, M. D., Edinburgh, Professor of Pathology in the Army Medical School, etc., etc., in two volumes, vol. 2. From the fourth London edition, with additions, by Meredith Clymer, M. D., late Professor of the Institutes and Practice of Medicine in the University of New York, etc. Philadelphia: Lindsay & Blakiston, 1866. For sale by Theodore Butler, Buffalo.

On the Action of Medicines in the System, by Frederick William Headland, M. D., B. A., F. L. S., Fellow of the Royal College of Physicians, etc., etc. Fifth American from the fourth London edition, revised and enlarged. Philadelphia: Lindsay & Blakiston, 1867. For sale by Theodore Butler, Buffalo.

Guide for using Medical Batteries; (being a compendium from his larger work on Medical Electricity and Nervous Diseases,) showing the most approved Apparatus, Methods and Rules for the Medical employment of Electricity in the treatment of Nervous Diseases. By Alfred C. Garratt, M. D., Fellow of the Massachusetts Medical Society, etc. Philadelphia: Lindsay & Blakiston, 1867. For sale by Theodore Butler, Buffalo.

Watson Abridged; a Synopsis of the Lectures on the Principles and Practice of Physic, delivered at King's College, London, by Thomas Watson, M. D., Fellow of the Royal College of Physicians, etc. Abridged from the last English edition, with a concise but complete account of the properties, uses, preparations, doses, etc., by J. J. Meylor, A. M., M. D. Philndelphia: Published by the Author. For sale by Theodore Butler, Buffalo.

Why Not? A Book for every Woman. The Prize Essay to which the American Medical Association awarded the Gold Medal for 1865. By Horatio Robinson Storer, M. D., Surgeon to the Franciscan Hospital for Women, etc. Boston: Lee & Shepard, 1867.

Medical Register of the District of Columbia, 1867. Embracing Notices of the Medical, Benevolent and Public Institutions of Washington, by J. M. Toner, M. D.

Code of Medical Ethics adopted by the American Medical Association, revised to date. New York: William Wood & Company, 1867.

A Theory of Inflammation; its Cause, Course, and Rationale of Treatment. By Nelson M. North, M. D., Surgeon Metropolitan Police, etc. New York: Wm. Wood & Company, 1867.

Report to the International Sanitary Conference, of a commission from that body, on the Origin, Endemicity, Transmissibility and Propagation of Asiatic Cholera. Translated by Samuel L. Abbott, M. D., Physician of the Massachuseits General Hospital, etc.

Advice to Students—An Address delivered at the opening of the Medical Lectures of Harvard University, Nov. 7, 1866, by Prof. C. E. Brown-Séquard, M. D.

Twenty-fourth Annual Report of the Managers of the New York State Lunatic Asylum, for the year 1866.

